# Supplementary figures and images for: UNBRANCHED3 Expression and Inflorescence Development is Mediated by UNBRANCHED2 and the Distal Enhancer, KRN4, in Maize
Source: PLoS Genet. 2020 Apr 24;16(4):e1008764. doi: 10.1371/journal.pgen.1008764 (PMC7202667; doi:10.1371/journal.pgen.1008764)

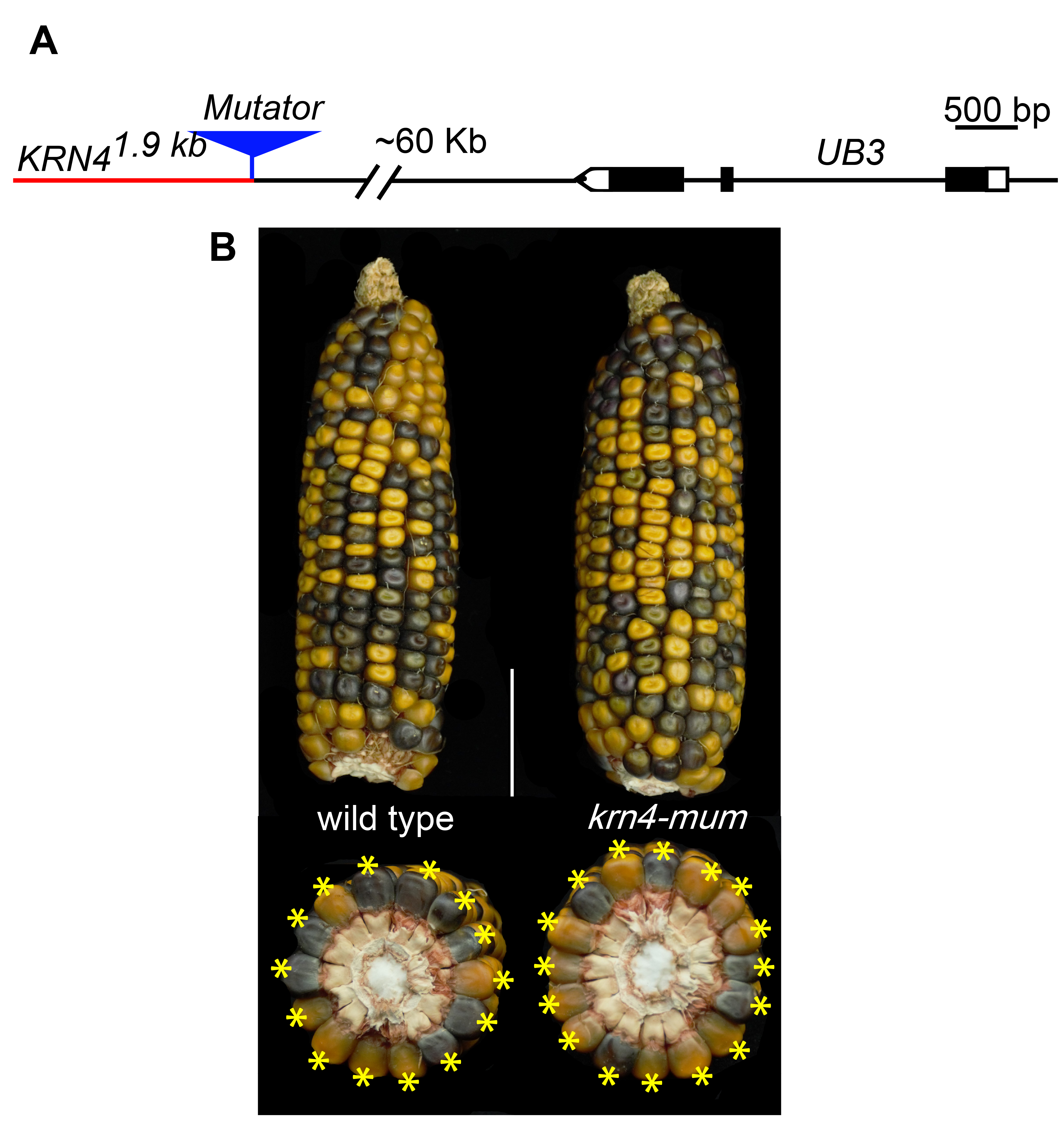

Supplement: S1 Fig — (A) Schematic diagram of the genomic locations of KRN4 and UB3 and the Mutator insertion site. Bar = 500 bp. Red line shows KRN4 locus. (B) Ears of wild type and krn4-mum. A represent ear of krn4-mum having 16 kernel rows (right) and a represent ear of wild type having 14 kernel rows (left) are shown. Bar = 4 cm. (TIF) [file pgen.1008764.s001.tif]

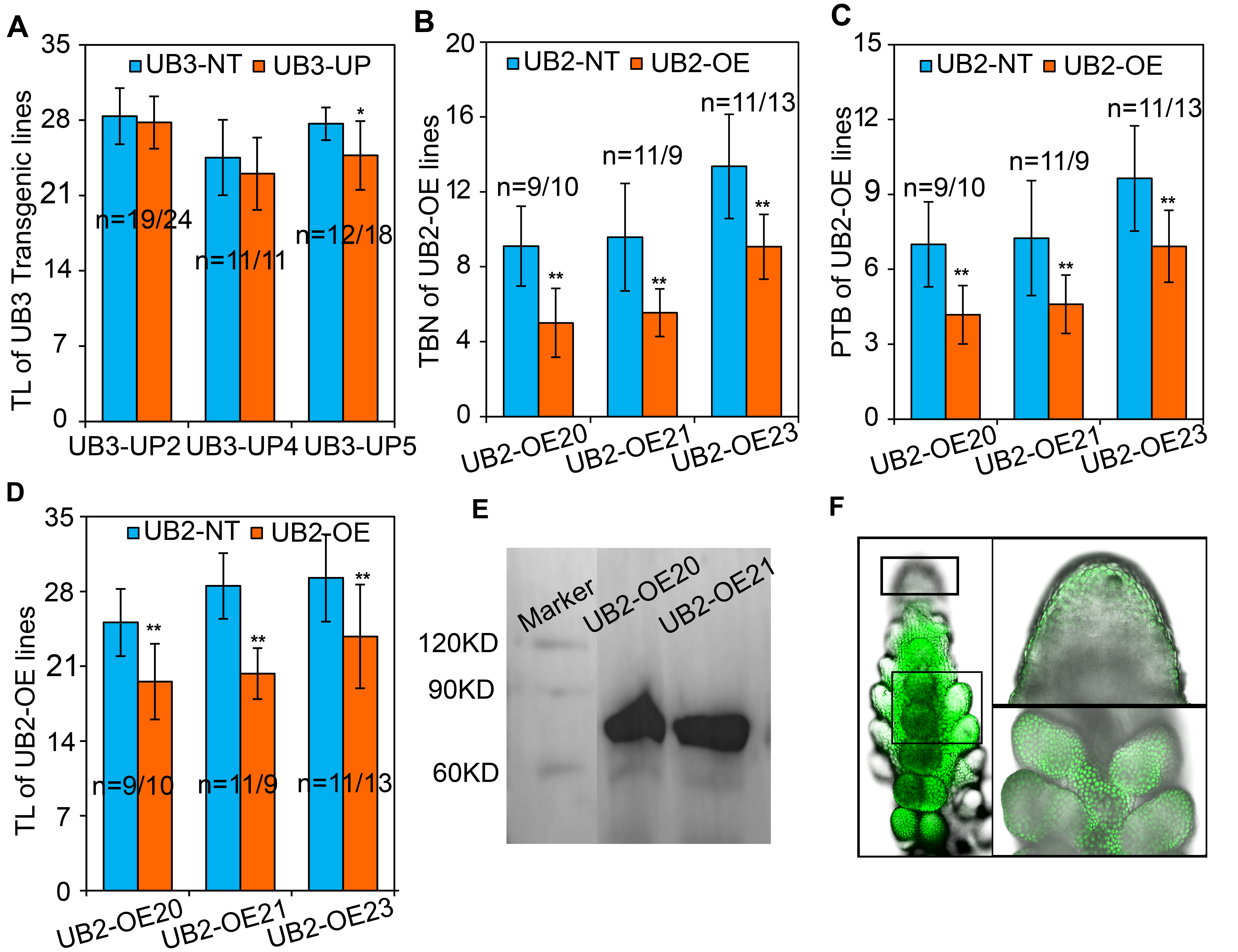

Supplement: S2 Fig — (A) Tassel length of the three independent UB3 transgenic lines (UB3-UP) and non-transgenic lines (UB3-NT). (B-D) Tassel branch and tassel length of UB2 transgenic plants (UB2-OE) and non- transgenic plants (UB2-NT). (E) Immunoblot analysis using YFP antibody. Proteins were extracted from the ears of UB2-OE20 and UB2-OE21 transgenic plants. (F) UB2-YFP fusion protein signals observed in 2 mm ears by confocal microscopy. Strong signals indicate that UB2-YFP fusion protein was expressed in the ears of the transgenic lines. n, number of individual tassel. Values are means ± SD, P-value was calculated by Student’s t-test. *, p<0.05; **, p<0.01. (TIF) [file pgen.1008764.s002.tif]

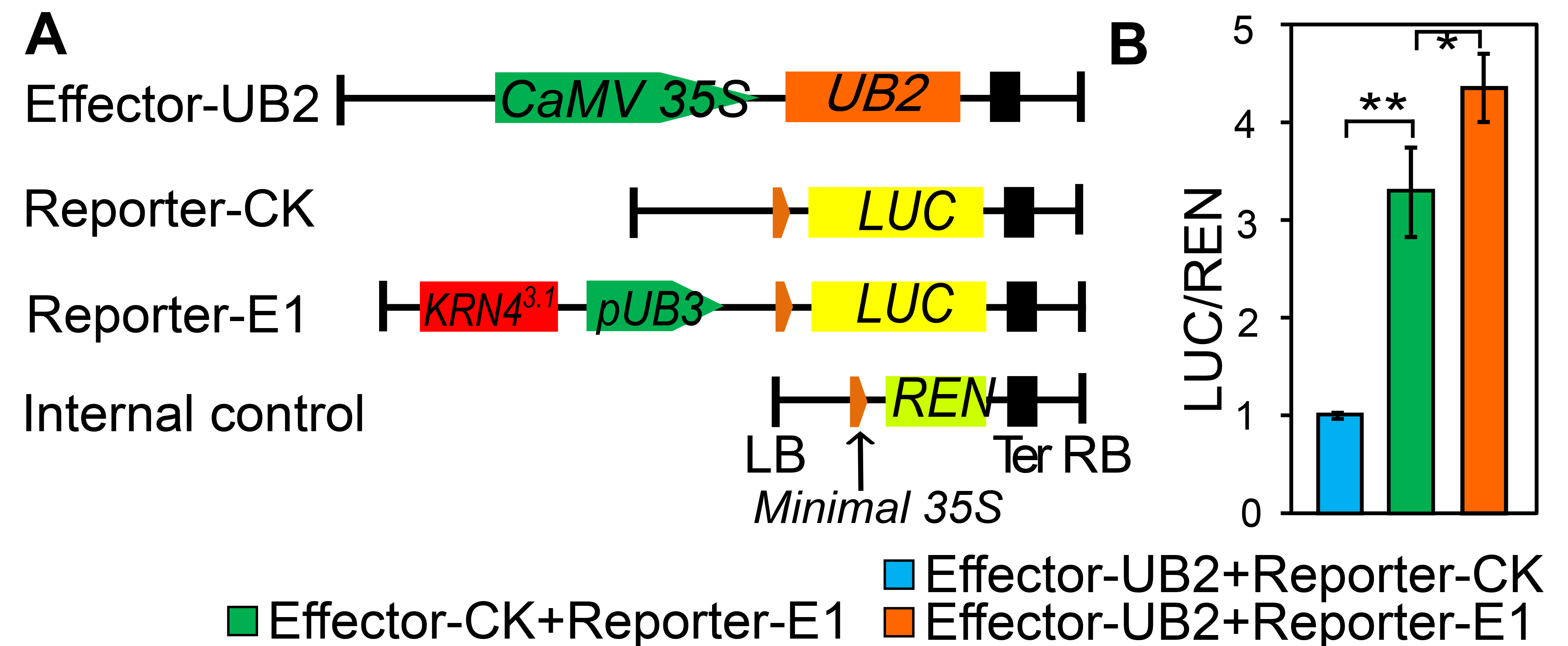

Supplement: S3 Fig — (A) Effector and reporter constructs. (B) Relative luciferase activity determined by transient expression analysis of KRN4-UB3 promoter-driven LUC in maize protoplasts co-infiltrated with UB2 effector. For each transient assay, 4–6 biological replicates and two technical replicates were performed. The value is presented as mean (LUC/REN) ± SD. ** P < 0.01; * P < 0.05. (TIF) [file pgen.1008764.s003.tif]

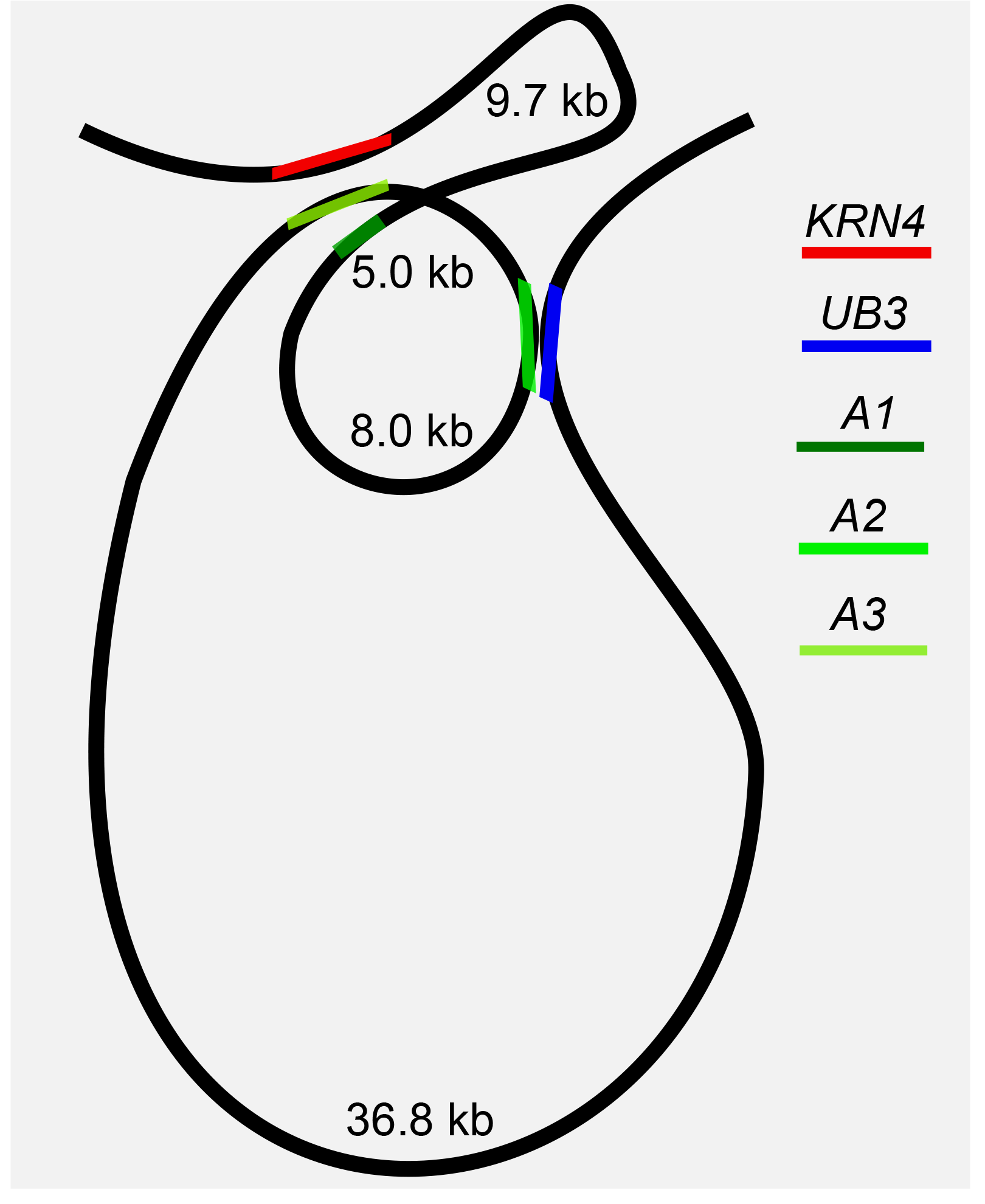

Supplement: S4 Fig — (TIF) [file pgen.1008764.s004.tif]

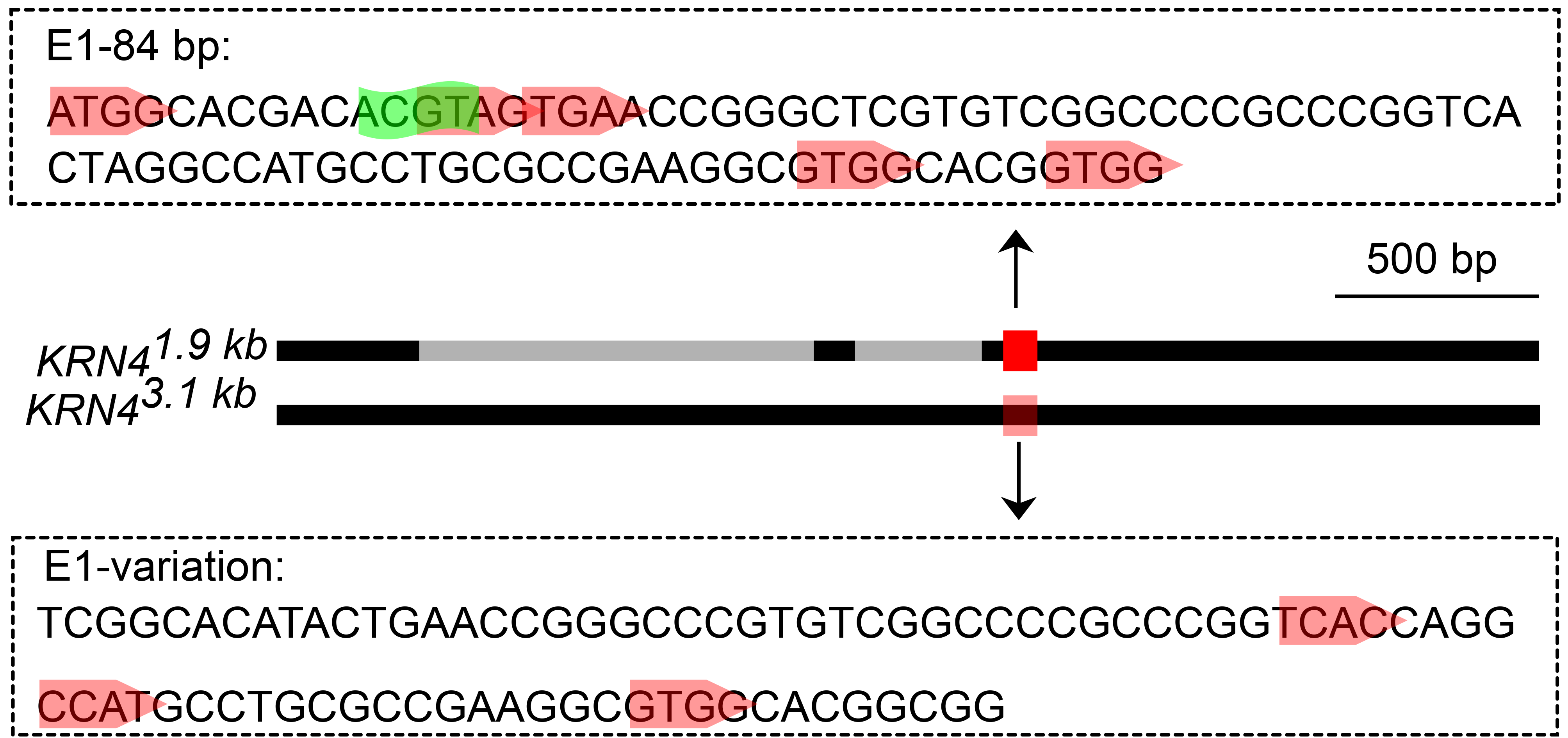

Supplement: S5 Fig — Sequences highlighted in red are enhancer-like elements. The sequence highlighted in green is the bZIP binding motif. (TIF) [file pgen.1008764.s005.tif]

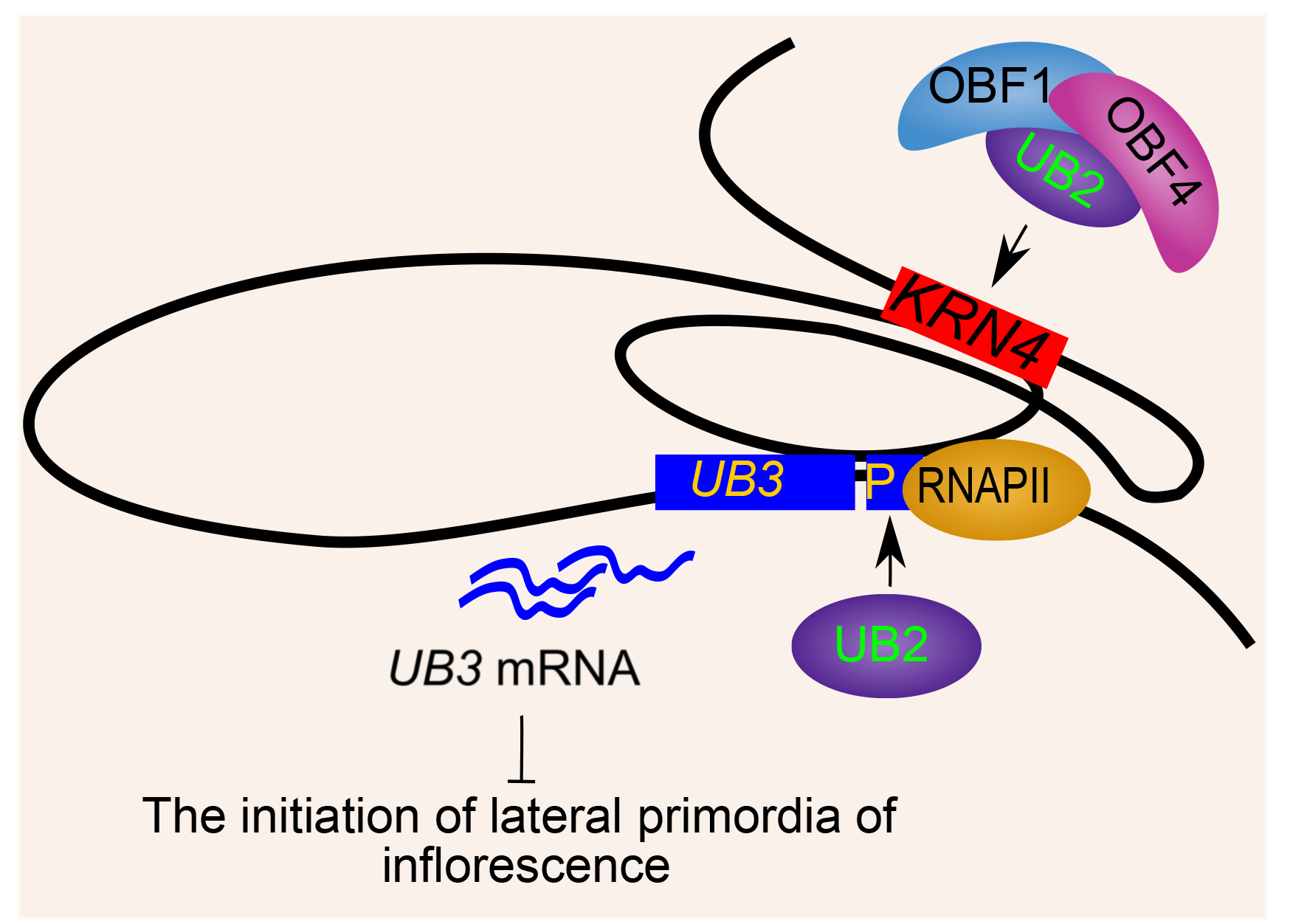

Supplement: S6 Fig — The two long-distant elements (KRN4, UB3 promoter) kept in close proximity to each other by chromatin interaction. Three interacting transcription factors UB2, OBF1 and OBF4 bind to specific cis-elements (GTAC motif or enhancer elements) harbored in UB3 promoter and KRN4 to promote the transcription of UB3 which negatively controls the initiation of reproductive axillary meristems and in turn the ear inflorescence branching. (TIF) [file pgen.1008764.s006.tif]
