# Supplementary material for: UNBRANCHED3 Expression and Inflorescence Development is Mediated by UNBRANCHED2 and the Distal Enhancer, KRN4, in Maize
Source: PLoS Genet. 2020 Apr 24;16(4):e1008764. doi: 10.1371/journal.pgen.1008764 (PMC7202667; doi:10.1371/journal.pgen.1008764)
Supplement: S1 Table — (DOCX) [file pgen.1008764.s007.docx]

S1 Table Probes used for EMSA

| Binding sites | Probe | Probe sequence （5' to 3'） |
| --- | --- | --- |
| 3×E1 repeat | 3E1X-F | ACATGGCACGACACGTAGTGAACCACATGGCACGACACGTAGTGAACCACATGGCACGACACGTAGTGAACC |
|  | 3E1X-R | GGTTCACTACGTGTCGTGCCATGTGGTTCACTACGTGTCGTGCCATGTGGTTCACTACGTGTCGTGCCATGT |
|  | 3E1XM-F | ACATGGCACGAACATGAGTGAACCACATGGCACGAACATGAGTGAACCACATGGCACGAACATGAGTGAACC |
|  | 3E1XM-R | GGTTCACTCATGTTCGTGCCATGTGGTTCACTCATGTTCGTGCCATGTGGTTCACTCATGTTCGTGCCATGT |
| KRN4-P4 | EP4-F | GCGTGTTGCCTACTTGCACTAGAGAGTACGTGAGGCAAACCAAATGTTTTCTAGCGAAC |
|  | EP4-R | GTTCGCTAGAAAACATTTGGTTTGCCTCACGTACTCTCTAGTGCAAGTAGGCAACACGC |
|  | EP4M-F | GCGTGTTGCCTACTTGCACTAGAGAGCACGTGAGGCAAACCAAATGTTTTCTAGCGAAC |
|  | EP4M-R | GTTCGCTAGAAAACATTTGGTTTGCCTCACGTGCTCTCTAGTGCAAGTAGGCAACACGC |
| pUB3-P1 | EPUB3-F | GCCGAGGGCTTTTTGTAGACGCCAGGTACTTGCGCTTGTGGCGGCGAGCCGAGCG |
|  | EPUB3-R | CGCTCGGCTCGCCGCCACAAGCGCAAGTACCTGGCGTCTACAAAAAGCCCTCGGC |
|  | EPUB3M-F | GCCGAGGGCTTTTTGTAGACGCCAGGCACTTGCGCTTGTGGCGGCGAGCCGAGCG |
|  | EPUB3M-R | CGCTCGGCTCGCCGCCACAAGCGCAAGTGCCTGGCGTCTACAAAAAGCCCTCGGC |
|  |  |  |
