# Supplementary material for: UNBRANCHED3 Expression and Inflorescence Development is Mediated by UNBRANCHED2 and the Distal Enhancer, KRN4, in Maize
Source: PLoS Genet. 2020 Apr 24;16(4):e1008764. doi: 10.1371/journal.pgen.1008764 (PMC7202667; doi:10.1371/journal.pgen.1008764)
Supplement: S2 Table — (DOCX) [file pgen.1008764.s008.docx]

S2 Table Primers used for vector construction and genotype identification.

| Primer name | Primer sequence (5' to 3') | Purpose |
| --- | --- | --- |
| Full-cdna F | GGGGTAACC ATGGAGGCCGGCGGCGCCA | Amplification of UB3 CDS |
| Full-cdna R | GGAGATCT CTAGAGCGACCACTCCGTCGT |  |
| YFP-F | GGAGATCT ATGGTGAGCAAGGGCGAGGA | Amplification of YFP CDS |
| YFP-R | GGAGATCT TGGCCCCAGCGGCCGCAGCA |  |
| Barzz-F | CACCATCGTCAACCACTAC | transgene vector identification |
| Barzz-R | CAGTTCCCGTGCTTGAAG |  |
| krn4-mum1-F | AGTGGGCTGTGATTTGGAAAGT | *krn4-mum* genotype identification |
| krn4-mum1-R | GGCCGTTTTATACTGTGAACTGAT |  |
| TIR6 | AGAGAAGCCAACGCCAWCGCCTCYATTTCGTC | degenerate primer |
| ub2-mum1-F | TAAAGAAGAGCTGCCGCAAACGCCT | *ub2* genotype identification |
| ub2-MUM1-R | CATAGTGGGCGCCGTATC |  |
| ub3-mum1-F | TGCTGGATTTCTCATACCCAAGG | *ub2* genotype identification |
| ub3-mum1-R | AGCCTCTAGGCAAGCAAACA |  |
| Mu9242 | AGAGAAGCCAACGCCAWCGCCTCYATTTCGTC | *Mu*-end primer |
| mp35S-F | ACCCCGGGATCTCCACTGACGTAAGGGATGACGCACAATCCCACTATCCTTCGCAA  GACCCTTCCTCTATATAAGGAAGTTCATTTCATTTGGAGAGGAAAGCTTAC | Minimal 35S cloned to PGL3-basic |
| mp35S-R | ACAAGCTTTCCTCTCCAAATGAAATGAACTTCCTTATATAGAGGAAGGGTC  TTGCGAAGGATAGTGGGATTGTGCGTCATCCCTTACGTCAGTGGAGATCCCGGGAC |  |
| recmp35S-2F | GCCTGTCGACGCGTAGAATTCATCTCCACTGACGTAAGGGA | Minimal 35S cloned to pRL-null |
| recmp35S-2R | CTGCAGTACCCGGGTGAATTCTCCTCTCCAAATGAAATGAA |  |
| recB3015pro_F | ATTTCTCTATCGATAGGTACCGACCGTTTTAGTGAAGTAGACC | UB3 promoter cloned to PGL3-basic |
| recB3015pro_R | CACGCGTAAGAGCTCGGTACCGTCTGTCTCCCTCTCTCTCAG |  |
| rec2NX41F | TGGTAAAATCGATAAGGATCCATTCTACGGAATGTCTTGGA | KRN4 cloned to PGL3-basic |
| rec2new42-R | AGGGCATCGGTCGACGGATCCTTAGTGGAGATAGAAAAGTT |  |
| rec2new41-F | TGGTAAAATCGATAAGGATCCTTAATACTTGCGTCATCTCT | KRN4 cloned to PGL3-basic |
| rec2new42-R | AGGGCATCGGTCGACGGATCCTTAGTGGAGATAGAAAAGTT |  |
| HES1-F | AATCGATAAGGATCCGTCGACATGGCACGACACGTAGTGAA | E1 cloned to PGL3-basic |
| HES1-R | CTCTCAAGGGCATCGGTCGACCCACCGTGCCACGCCTTCGG |  |
| RTLOBF1-F | GGTACCCGGGGATCCTCTAGAATGTCGTCGTCGTCGCTGTC | OBF1 CDS cloned to pRTL2 |
| RTLOBF1R | GTGATTTTTGCGGACTCTAGATCAGTAGTGGAGCATGTGCG |  |
| RTLOBF4-F | GGTACCCGGGGATCCTCTAGAATGGAGAGTAGACGGGGAGG | OBF4 CDS cloned to pRTL2 |
| RTLOBF4-R | GTGATTTTTGCGGACTCTAGATTATTCCCTTGGACGGGCAA |  |
| RTLUB2-F | GGTACCCGGGGATCCTCTAGAATGGAGTCCGGCGGTGGCGG | UB2 CDS cloned to pRTL2 |
| RTLUB2-R | GTGATTTTTGCGGACTCTAGACTAGAGCGACCAGTCCATCG |  |
| ADOBF1-F | GCCATGGAGGCCAGTGAATTCATGTCGTCGTCGTCGCTGTC | OBF1 CDS cloned to pGADT7 |
| ADOBF1-R | CAGCTCGAGCTCGATGGATCCTCAGTAGTGGAGCATGTGCG |  |
| ADOBF4-F | GCCATGGAGGCCAGTGAATTCATGGAGAGTAGACGGGGAGG | OBF4 CDS cloned to pGADT7 |
| ADOBF4-R | CAGCTCGAGCTCGATGGATCCTTATTCCCTTGGACGGGCAA |  |
| ADUB2-F | GCCATGGAGGCCAGTGAATTCATGCGCTGCCAGGTCGACGGCTG | UB2 CDS cloned to pGADT7 |
| ADUB2-R | CAGCTCGAGCTCGATGGATCCCTAGAGCGACCAGTCCATCG |  |
| phisE1-F | ATTCCCGGGGAGCTCACGCGTATGGCACGACACGTAGTGAA | E1 cloned to pHISi-1 |
| phisE1-R | CGGATCGATTCGCGAACGCGTCCACCGTGCCACGCCTTCGG |  |
| 4TUB2-F | CCGCGTGGATCCCCGGAATTCATGCGCTGCCAGGTCGACGGCTG | UB2 CDS cloned to pGEX-4T-1 |
| 4TUB2-R | GTCACGATGCGGCCGCTCGAGCTAGAGCGACCAGTCCATCG |  |
| MBPOBF1-F | GAGGGAAGGATTTCAGAATTCATGTCGTCGTCGTCGCTGTC | OBF1 CDS cloned to pMal-c2X |
| MBPOBF1-R | ACGACGGCCAGTGCCAAGCTTTCAGTAGTGGAGCATGTGCG |  |
| MBPOBF4-F | GAGGGAAGGATTTCAGAATTCATGGAGAGTAGACGGGGAGG | OBF4 CDS cloned to pMal-c2X |
| MBPOBF4-R | ACGACGGCCAGTGCCAAGCTTTTATTCCCTTGGACGGGCAA |  |
| 771-OBF1F | CGAGCTCGGTACCCGGGATCCATGTCGTCGTCGTCGCTGTC | OBF1 CDS cloned to JW771 |
| 771-OBF1R | CGCGTACGAGATCTGGTCGACGTAGTGGAGCATGTGCGGGG |  |
| 771-OBF4F | CGAGCTCGGTACCCGGGATCCATGGAGAGTAGACGGGGAGG | OBF4 CDS cloned to JW771 |
| 771-OBF4R | CGCGTACGAGATCTGGTCGACTTCCCTTGGACGGGCAAGCC |  |
| 771-UB2F3 | CGAGCTCGGTACCCGGGATCCATGCGCTGCCAGGTCGACGGCTG | UB2 CDS cloned to JW771 |
| 771-UB2R3 | CGCGTACGAGATCTGGTCGACGAGCGACCAGTCCATCGTGT |  |
| 772-OBF1F | TACGCGTCCCGGGGCGGTACCATGTCGTCGTCGTCGCTGTC | OBF1 CDS cloned to JW772 |
| 772-OBF1R | ACGAAAGCTCTGCAGGTCGACTCAGTAGTGGAGCATGTGCG |  |
| 772-OBF4F | TACGCGTCCCGGGGCGGTACCATGGAGAGTAGACGGGGAGG | OBF4 CDS cloned to JW772 |
| 772-OBF4R | ACGAAAGCTCTGCAGGTCGACTTATTCCCTTGGACGGGCAA |  |
| 772-UB2F3 | TACGCGTCCCGGGGCGGTACCATGCGCTGCCAGGTCGACGGCTG | UB2 CDS cloned to JW772 |
| 772-UB2R3 | ACGAAAGCTCTGCAGGTCGACCTAGAGCGACCAGTCCATCG |  |
|  |  |  |
