# Supplementary material for: UNBRANCHED3 Expression and Inflorescence Development is Mediated by UNBRANCHED2 and the Distal Enhancer, KRN4, in Maize
Source: PLoS Genet. 2020 Apr 24;16(4):e1008764. doi: 10.1371/journal.pgen.1008764 (PMC7202667; doi:10.1371/journal.pgen.1008764)
Supplement: S3 Table — (DOCX) [file pgen.1008764.s009.docx]

S3 Table Primers used for quantitative RT-PCR and *in situ*.

| Gene ID or Genome Chromatin sites (B73-V4) | Primer name | Primer sequence (5' to 3') | Purpose |
| --- | --- | --- | --- |
| *Zm00001d052890* | 30qanti3-F | GCAACAGCAGGCTTCGAC | QRT-PCR for *UB3* |
|  | 30qanti3-R | ACCTGAGAACTGGCCGTGAT |  |
| *Zm00001d010159* | qACTIN-F | TACGAGATGCCTGATGGTCAGGTCA | internal control of QRT-PCR |
|  | qACTIN-R | TGGAGTTGTACGTGGCCTCATGGAC |  |
| *Zm00001d031451* | SBP917_F | ACCCCTTGCGTCACGATAC | QRT-PCR for *UB2* |
|  | SBP917_R | GTACGAGAAATCCAGCATGA |  |
| *Zm00001d030617* | I-OBF1_F | GACATCGCGTCCCAGTACAC | *In situ* for *OBF1* |
|  | I-OBF1_R | CCAGATCTGAATAAACGCTTGA |  |
| *Zm00001d012553* | I-OBF4_F | TGCTGGTGATAATGATCTTCGT | *In situ* for *OBF4* |
|  | I-OBF4_R | ACTGGTTTGTTCGTCCATCATA |  |
| *Zm00001d052890* | I-UB3_F | GTTCAGAAGCTTCATGCTGGAT | *In situ* for *UB3* |
|  | I-UB3_R | CAACATTAGCGGAAGGATGAG |  |
| *Zm00001d031451* | I-UB2_F | ATGCGCTGCCAGGTCGACGGCTG | *In situ* for *UB2* |
|  | I-UB2_R | CTAGAGCGACCAGTCCATCG |  |
| Chr4:203547380..203547611 | KRN4-P1F | AGTCCATGAGTGGCTCACTCAC | ChIP-qPCR for KRN4-P1 |
|  | KRN4-P1R | TCAGGAGAGCAAGAGAGATGTC |  |
| Chr4:203548224..203548497 | KRN4-P2F | CGGCGGCAAGGCAACTTCTTCTG | ChIP-qPCR for KRN4-P2 |
|  | KRN4-P2R | CCAAGTCCCACGAGGTCGCCTC |  |
| Chr4:203549852..203550087 | KRN4-P3F | TGCGTCGGCACCACAGGTGGCG | ChIP-qPCR for KRN4-P3 |
|  | KRN4-P3R | ACTCTTCCCTCACATCGAACGG |  |
| Chr4:203550330..203550433 | KRN4-P4F | GAAAGTTGCGTCGCCACCGA | ChIP-qPCR for KRN4-P4 |
|  | KRN4-P4R | CATGATATCATTCGTCGCGGT |  |
| Chr4:203615692..203615893 | PUB3-P1F | TAGCTAGCCACGCCGCGGCG | ChIP-qPCR for PUB3-P1 |
|  | PUB3-P1R | TCCGCCTCGCTCGGCTCGCC |  |
| *Zm00001d013154* | NB149 | AGGAGCCTCACGCAGATAAA | Internal control |
|  | NB150 | CCTGCGGTAGAGGATGTTGT |  |
