# Supplementary material for: UNBRANCHED3 Expression and Inflorescence Development is Mediated by UNBRANCHED2 and the Distal Enhancer, KRN4, in Maize
Source: PLoS Genet. 2020 Apr 24;16(4):e1008764. doi: 10.1371/journal.pgen.1008764 (PMC7202667; doi:10.1371/journal.pgen.1008764)
Supplement: Attachment — Submitted filename: PG-response R1.doc [file pgen.1008764.s010.doc]

Response to reviewers’ comments

Reviewer #1: Maize kernel row number is an important agronomical trait that influences grain yield. The genes UB2, UB3 and KRN4 locus are key components regulating kernel row number and inflorescence development. Some previous studies have indicated that these components genetically interact with each other. However, it is still unclear how these components work together to regulate inflorescence development. In the present manuscript, Du et al. investigated the genetic and molecular effects of KRN4 with UB2 and UB3 to reveal the mechanism whereby KRN4 fine-tunes UB3 expression and, thus, inflorescence development. This work is of interest for the readership and is suitable to be published in PLOS Genetics after minor revision.
Major concerns:
1. On the figure 6D (line 683), the authors used Reporter-CK construct as control, that's very inappropriate! They should use “Reporter-E1” as control and use “Reporter-E1 + Effector-OBF1 or Effector-OBF4” as experimental group, because as shown in Fig 5B, Reporter-E1 itself shows much higher LUC activity than Reporter-CK.
The same problem occurs in Fig S3 (line 728).
Response: Thanks for the comments.

We agree with this suggestion. In the updated version, we use both “Effector-OBF1/OBF4/UB2 + Reporter-CK” and “Effector-CK + Reporter-E1”as negative controls, and “Reporter-E1 + Effector-OBF1 or Effector-OBF4 or Effector-UB2” as experimental groups in Fig 6C-6D (P28 Line749), also in S3 Fig (Sup-P3 Line35)

2. The results in the manuscript show that UB2, OBF1 and OBF4 can directly bind to KRN4 region (Fig 4G and 6A-B), and each of them can elevate the KRN4-mediated enhancement effect on downstream gene expression (Fig 6D and S3). It is of interest whether these three TFs show synergetic effect on KRN4-mediated expression of downstream genes, such as UB3. I suggest the authors investigate this synergetic effect by the combination of three TFs using transient expression assays.
Response: This is a good idea. We are also interested in the synergetic effect of these three factors in maize protoplast transient expression system. We had repeatedly performed many attempts, and found that most of the protoplasts broken down after overnight culture, which led to the signals of both firefly luciferase and Ren luciferase are very weak, showing the co-transformation of multiple transcription factors strongly impacts on normal development, growth and survival of protoplasts. Thus, transient expression system might not be an effective way for detecting the synergetic effect of the combination of three TFs. Hopefully, we plan to use “phase separation model” to explore transcription control of super enhancers, which will helps to elucidate transcription factors UB2, OBF1, OBF4 and others enriched in the KRN4-UB3 chromatin interaction interval in the phase-separated droplet.

Minor concerns:
1. Fig 6A, the yeast clones growing on 0 mM 3-AT plate are smaller than those in 20 mM 3-AT and 30 mM 3-AT, a scale bar needs to be added.
Fig 6B, the shifted bands are not in line with the free probes, it seems that they are not derived from the same membrane.
Response: Thanks. We added a scale bar in the updated Fig 6A (P28 Line749), and added “Bar = 0.3 cm” in the figure 6 legend (P28 Line752).

The bands in the Fig 6B are derived from the same membrane. The bands on the gel were likely curved when they were transferred to the nylon membrane.

2. Line 696, “KRN4 binding proteins”, instead of “KRN4 binging proteins”.
Response: We corrected it with “mRNA Expression patterns of *KRN4* binding proteins” (P28 Line762)

3. Line 755, Table S1, it’s better to underline the mutant sites in 3×E1 repeats, KRN4-P4 and pUB3-P1 sequences.

Response: In the updated version, we underline both the motif sequence and the mutant sites in Table S1 (Sup-P7 Line 59).

Reviewer #2: The authors previously have revealed that KRN4 controls quantitative variation in maize kernel row number, and UNBRANCHED3 regulates branching by modulating cytokinin biosynthesis and signalling in maize. In the present study, the authors provide experimental evidence for genetic interactions between KRN4 and UB3 as well as UB2 in controlling inflorescence architecture, enhancer activity of KRN4 toward UB3 promoters, and KRN4 recruited UB2-centred transcription complex for UB3 transcription. The findings provide new insights for improving KRN and yield in maize. The authors need address the following points before it could be accepted for publishing in PLoS Genetics.

1. The manuscript needs thoroughly language editing before accepting for publication.

Response: Thanks. The updated manuscript has been thoroughly edited by China Lunde in the USDA Plant Gene Expression Center, USA.

2. Line 165, UB2 transcription “levels” exhibited >8-fold increases, “and” UB3…

Response: Thanks. The sentence is revised as follow:

“In all three transgenic lines, *UB2* transcription levels exhibited >8-fold increases, and *UB3* transcription levels were also increased by 1–2-fold relative to the respective nontransgenic lines (UB2-NTs) (Fig 4B).” (P7 Line181)

3. Line 351, should be “Total RNA” while “mRNA” in Line 355

Response: Thanks. The sentence is revised as follow:

“Total RNA was isolated from the tissues of ~2 mm and 5 mm ears of various maize plants, cDNA was synthesized from mRNA using an…” (P14 Line 390, P14 Line394)

4. Line 618, the reference is not properly numbered.

Response: Thanks. We numbered the reference with “58”. (P22 Line683)

5. Figure 1,
a) Please specify what the red lines stand for
b) Figure legends required for the bar charts
c) Is the expression level calculated in relative to blue bars? Please clarify.
d) No significance at P < 0.05 level so the figure legends to be rephased

Response: Thanks. We added the description on the statistical analysis used, p-value, SD, red line and asterisk. The updated legend of figure1 is as follow:

“(A) Schematic diagrams of *KRN4* alleles (*KRN4^3.1^* and *KRN4^1.9^*) in the H21 genetic background. (B) Schematic diagrams of *KRN4* alleles (*krn4-mum* and *KRN4^1.9^*) in the W22 genetic background. The red line stands for *KRN4* locus, the triangle indicates the inserted *Mutator* element, bar = 3 kb. (C, D) *UB3* expression levels in 2-3 mm ears of near-isogenic lines in the H21 (C) and W22 (D) genetic backgrounds using quantitative PCR, with three biological replicates in each case. The values show the fold changes. (E, F) Kernel row number of near-isogenic lines in H21 (E) and W22 (F) genetic backgrounds. Values are shown as the mean ± s.d. Comparsion with the line with *KRN4^1.9^*, expression level of *UB3* was significantly lower but kernel row number was significantly higher in the line with *KRN^3.1^* or *krn-mum*. The *P*-value was calculated by one-way ANOVA. **, p<0.01. n: number of individual ears.”(P23 Line689-697)

6. Figure 2
a) Again, how is the expression data calculated or normalised? Error bar means SD or SE?
b) Is inflorescence length and number affected by UB3 expression?
c) In the subpanel H, please clarify the images with enlarged zones and add annotations to the red arrows.

Response: Thanks.

a) The expression data was using maize *ACTIN* (*Zm00001d010159*) gene as internal control; Error bar means SE. In figure2 legend we added “Values are shown as the mean ± s.e.” (P24 Line703-704)

b) Fig.2C (P24 Line 699) showed *UB3* expression has negative effect on tassel branch number (P6 Line132-135), we also analyzed the tassel length in *UB3* transgenic lines which also showing negative effect (S2A Fig) (Sup-P2 Line 21). In the updated version, the sentence is rephrased as follow:

“Similarly, reduced kernel row number, TBN and tassel length (TL) were observed in UB3-UP2 and UB3-UP5 compared with UB3-NT2 and UB3-NT5, respectively (Fig 2B-2D, S2A Fig), indicating negative regulations of inflorescence branches and length by *UB3*.” (P6 Line 135-138)

c) We clarified the images with enlarged zones and added explanatory notes on the “IM” and “SPM”. (P24 Line 699)

7. Figure 3
a) Why the ratio is set at 1.2 to define a fascinated ear? What is the rate of the representative cone in A? The image would cause misleading if the value is not 1.2.
b) A comparison of cross sections of the ear tips among mutants will be more intuitionistic.
c) The authors proposed the increased proportion of fascinated ears in krn4;ub2 is associated with the significant inhibition of UB3 transcription. The results showed a coordinated role of KRN4 and UB2 in the regulation of UB3 transcription, however, the fascination frequency could not match the UB3 expression since the krn4;ub2;UB3 had the highest rate but not lowest gene expression. Please explain.

Response: Thanks.

a) That is a good question. The ratio 1.2 is the statistics results of the d1/d2 in the wild type ear. The cross sections in “Fig.3A” (Line656) coming from a fasciated ear is to show a classical widest and narrowest diameters of the ear tip, the rate of d1/d2 is 2.2. We added a picture of the entire ear and marked the cut to make it more readable in Fig 3A (P25 Line 712).

b) The comparison of cross sections of the ear tips among mutants is a good idea. We replace the entire ears with ear tips and cross sections in Fig 3B (P25 Line 712).

c) We want to show the kernel row number or ear fascination in this case is caused by the change of *UB3* expression level. The results in this study and previous study (Chuck et al., 2014 and Liu et al., 2015) support that *UB3* negatively regulates kernel row number or ear fascination. So we propose the increased proportion of fasciated ears in *krn4;ub2* is associated with the significant inhibition of *UB3* transcription. However, our previous study also showed that *UB3* fine-tune rice tiller number and panicle branches in rice (Du et al., 2017). When *UB3* was highly expressed in rice, the tillers and branches were depressed; when *UB3* was moderately expressed, the number of panicle branches was increased (Du et al., 2017). Thus, the ear of *krn4;ub2;UB3* individual with moderate *UB3* expression might show highest rate of fascination frequency. In addition, genetic compensation and redundancy may help to explain the ear of *KRN4;UB2;ub3* individuals has the lowest *UB3* gene expression but almost of them show a normal ear tip.

8. Figure 4
a) It seems that the tassel size is significantly reduced in the OE lines, thus any phenotyping is conducted?
b) Please add colour legend to D.
Response: Thanks.

a) In UB2 OE lines, the tassel length and tassel branch number were significantly decreased (Fig 4C-4D; S2B-2D Fig). We added the data of tassel length in S2 Fig (Sup-P2 Line21-26), and we rephrased the sentence in this updated version as follow:

“Additionally, the UB2-overexpressing lines showed significantly reduced kernel row number, TBN and TL relative to the respective UB2-NT lines (Fig 4C-4D; S2B-2D Fig).” (P7 Line183-184)

b) We added the color legend in Fig.4D as followed:

“The blue and orange columns represent *UB2* non-transgenic and overexpressed lines, respectively.” (P26 Line726-727)

9. Figure 6, after how many days the yeast culture is photographed? The OBF1 binding activity seems very low as observed from the colony. Please explain.

Response: Thanks.

Yeast cells were cultured at 30℃ for 3 days and then at room temperature for 3 days, then photographed.

The binding activity of OBF1seems lower, one of the possible reasons is that OBF1 functions as a dimer or multimer with other co-factors. It has been reported that OBP (OBF binding protein) specifically increased the binding of the OBF proteins to ocs element sequences in Arabidopsis (Zhang B et al., 1995), so we suggested that specific OBPs in maize are required for the binding activity of OBF1. In this study, OBF1, OBF4 and UB2 can interact with each other and overlapped mRNA expression in maize immature ear, so they might form transcription complex to function well.

10. Figure 7, the figure has missing subpanels according to the legends so please add the relevant images. Line 702, should be “NP and CP” but not PN and PC.

Response: The “(J)” and “(K)” should be “(H)” and “(I)” (P29 Line765-766); “PN and PC” were replaced by “NP and CP” (P29 Line768)

11. 'L34 A large proportion of the plant genome consists of intergenic regions that were previously thought to lack biological functions'. This is an outdated statement and should not be in the Author summary

Response: Thanks. We removed it from the Author summary. (Line36)

Reviewer #3: Du et al. described a series of experiments to investigate the regulation of UB3 expression by KRN4, which is an intergenic region ~60 kb away. I feel that all relevant genetic materials were carefully generated, and experiments were done appropriately. The topic is relatively new, and the overall study is novel. The findings from this mechanistic study are very interesting to a broad range of scientists. I enjoyed reading the manuscript.

Only some minor comments.
1. The current discussion is narrow. It may be good to add a few general statements in Discussion to explain that “cis-regulatory element” still be “long-distance” and “distal”, similar to what you have in L81-86. And a brief discussion of other known cases?

Response: Thanks for your suggestions. In the introduction, we gave many examples of distal regulation like *qSW5/GW5* in rice, *BOOSTER1* (*B1*), *ZmRap2.7* and *TEOSINTE BRANCHED1* (*TB1)* in maize (P4 Line65-77).

Additionally, the “cis-regulatory element” is defined in the introduction section as follow.

“The cis-regulatory elements that are a key class of noncoding DNA sequences and act to regulate the transcription of a neighboring gene.” (P5 Line86-88)

2. Authors may discuss the genetic effect of the original mapping of KRN4 for kernel row number and the current findings. It is known that materials used for mechanistic study are often different from the mapping study. But with the currently proposed model, it would be interesting to try to explain the situation in the original mapping and cloning study.

Response: Thanks. We added the discussion on genetic effects of *KRN4* in the original mapping in this study as follow:

“Two parents used in *KRN4* mapping, H21 which has *KRN4^1.9^* allele and H21^NX531^ which has *KRN4^3.1^* allele, show significantly difference in kernel row number and *UB3* expression level [27]. In this study, we found that *KRN4^1.9^* in H21 exhibits a stronger enhancer activity than *KRN4^3.1^* in regulating *UB3* expression level that is negatively correlated with kernel row number. The result will help to understand the *KRN4* effects on *UB3* expression level and kernel row number in the original mapping population.”(P11 Line314-320)

3. I found a few issues in Fig. S1, which was supposed to support Fig. 1. While authors showed the picture of kernel row number of 14 for wild type and 16 for krn4-mum, the actual average numbers were roughly 14 and 14.5(?). Yes, it is significantly different, but authors need to point out that what was shown for krn4-mum (16) can only be observed from some of the ears. Authors need to point out this in the main text too.
Fig. S1C, something must be wrong for the right two bars (Sanya), if we believe what is show on the left two bars (Wuhan) is correct. It is not possible to obtain a p=0.03 if the standard deviation bars are so wide and the sample size (87/88) is only slightly higher than the left two bars (Wuhan, 76/84). On the other hand, it is likely that the left two bars are wrong, so are the Fig. 1F. The standard deviation bars should be wider that what is shown if you have row numbers of 12, 14, and 16. If authors figured out it is not significantly different for Sanya, this needs to be noted in Fig. S1, since only the results from Wuhan is in Fig. 1F.
In Fig. 1 “One-way ANOVA” was mentioned, but “Student’s t-test” was mentioned in Fig. S1. Please be consistent in description and analysis. Regardless whether these two terms mean the same thing in your case or give the same results. Check all other places.
Please inspect the raw data and analyses to make sure the calculations and plotting are all correct. Also, reviewing the difference between standard deviation and standard error would be helpful.
Please revise so that the color scheme is consistent with Fig. 1. For Fig. S1A, either remove the label “krn4-mum”, or move it up a bit to avoid confusion.

Response: Thank you for your careful review.

We re-analyzed the phenotype data (Sanya) and removed it (Sup-P1 Line14). In the updated version, we redraw Fig. S1 and rewritten sentence:

“The *UB3* expression in *krn4-mum* individuals was lower than that in *KRN4^1.9^* homozygotes (*p* = 0.003), and the kernel row number of *krn4-mum* (14.93±0.18) individuals was significantly higher than that of *KRN4^1.9^* individuals (14.36±0.36) (*p* = 0.03) (Fig 1D and 1F; S1A-B Fig)” (P6 Line118-121)

“A represent ear of *krn4-mum* having 16 kernel rows (right) and a represent ear of wild type having 14 kernel rows (left) are shown.” (Sup-P1 Line776-777)
